# Pine plantations and five decades of land use change in central Chile

**DOI:** 10.1371/journal.pone.0230193

**Published:** 2020-03-13

**Authors:** Sandra V. Uribe, Cristián F. Estades, Volker C. Radeloff

**Affiliations:** 1 LEVS, Departamento de Gestión Forestal y su Medio Ambiente, Universidad de Chile, La Pintana, Santiago, Chile; 2 SILVIS Lab, Department of Forest and Wildlife Ecology, University of Wisconsin-Madison, Madison, WI, United States of America; Universidad Miguel Hernandez de Elche, SPAIN

## Abstract

The expansion of forest plantations is cause for concern because of their environmental effects, and the loss of native forests and agricultural land. Our goal was to quantify the increase in pine plantation, and concomitant loss of native forests, in central Chile since ca. 1960, and to identify in which settings native forests were lost most rapidly. We analyzed aerial photographs from 1955 and 1961, Landsat images from 1975 and 1998, and Google Earth high-resolution satellite images from 2014. To ensure high classification accuracy, we visually interpreted images for a systematic 3-km grid and assigned each point as either ‘pine plantation’, ‘native forest’, ‘agricultural-livestock lands’, or ‘other’. We also calculated latitude, longitude, slope, Euclidean distance to the nearest road and to the nearest pulp mill, and the frequency of land use surrounding each point as potential variables to explain observed land use changes. Pine plantations expansion started even before 1960, when 12% of all points were already pine plantations, was particularly rapid from 1975 (18% of sample points) to 1998 (38%), and stabilized thereafter (37% by 2014). From 1975 to 1998 alone, 40% of native forests were replaced by pine plantations, and agricultural-livestock lands declined by 0.7%, 0.9%, 1% per year before 1975, from 1975 to 1998, and after 1998 respectively. Native forests that were surrounded by pine plantations, were most likely to be converted to plantations, and from 1960 to 1975, also native forests near pulp mills. The probability of change from agricultural-livestock lands to pine plantations was mainly influenced by slope, with most agricultural-livestock lands remaining in areas with low slopes.

## Introduction

Native forest are rapidly lost world-wide with dire consequences for biodiversity [1–3]. One of the most important causes of native vegetation loss is land use and land cover change (LULCC) [4–6] affecting biodiversity directly due to habitat fragmentation, biotic homogenization, ecosystem services loss, and other factors [7–9].

While natural forests have declined between 1990 and 2015 from 3961 M ha to 3721 M ha [10] in many developing countries, forest area is increasing, partly due to natural forest regrowth on abandoned agriculture, and partly due to plantations [11–13]. The forest transition hypothesis proposed by Mather and Needle [14] suggests that the concentration of agricultural production by farmers on better soils, promotes the abandonment of poorer soils, and allowing for natural reforestation or plantations, thereby increasing forest cover in some places [15,16]. An important forest cover increase, after agricultural abandonment, is in the form of forest plantation expansion [13,17], and that has been observed in several developing countries, mainly in Asia and Latin America [18–20].

While worldwide natural forest declined between 1990 and 2015, forest plantations increased from 168 M ha to 278 M during the same period [10]. Plantations are often promoted by governments to bring abandoned or agricultural unproductive lands back into use, and mechanisms for that promotion include tax reductions or subsidies with the purpose of stimulating economic development and forestry industry [21,22]. Nevertheless, not only abandoned and unproductive lands have been converted into plantations, native vegetation has also been transformed into forest plantations (e.g. [23–25]), which means that the growing of global forest cover entails concomitant native forest loss (e.g. [19,26]).

Agglomeration economies (sensu [27]) may be important, but their effects for forest plantations are largely unclear. Socio-economic forces affect land use change greatly, especially when industrial centers are placed in rural localities, which changes people interactions, including the way they carry out their productive activities, and their migration to cities [22, 28,29]. Furthermore, a clustering process of industrial activities occurs when this is profitable, promoting the development of the industry in adjacent lands to be transformed in the same production activity due to agglomeration effects [29,30]. Deforestation has been promoted by such pressures, especially where there is a lack of legal and political strategies to prevent it [30–32].

As has been the case throughout most of the world, forest plantations in Chile were promoted with the aim to protect and use soils that were highly damaged due to intensive agricultural production [33]. In 1931, the Law Decree N°4363 was enacted, being the first Forest Law in Chile [34]. This Law included both native forest protection, and the protection of soils via tree plantations, with tax exemptions and financial rewards for land owners who planted trees, and other assistances for planting [35]. Over time, as forest plantations proved to be highly profitable, economic and political pressures favored their expansion into all types of land cover, not just degraded lands. Studies that relate land use change with the increase of forest plantation in Chile reveal an important transformation of native vegetation into plantations since the 1970s (e.g. [6,23,24]), which coincides with the enactment of Law Decree 701 in 1974, whose main purpose was to subsidy reforestation. Most of the studies that have focused on historical LULCC in Central Chile, start their analysis in the 1970s, when the first satellite images were recorded (e.g. [2,23,36]). However, Monterey pine plantations were already widespread in this region during the 1950s, and even before [34,37,38], making it difficult to ascertain if pine plantations replaced native forests or agricultural land.

Our goal here was to quantify the increase in pine plantations, and concomitant loss of native forests, in central Chile since ca. 1960, and to identify the conditions in which native forests were lost most rapidly.

## Methods

### Study region

**Fig 1:**
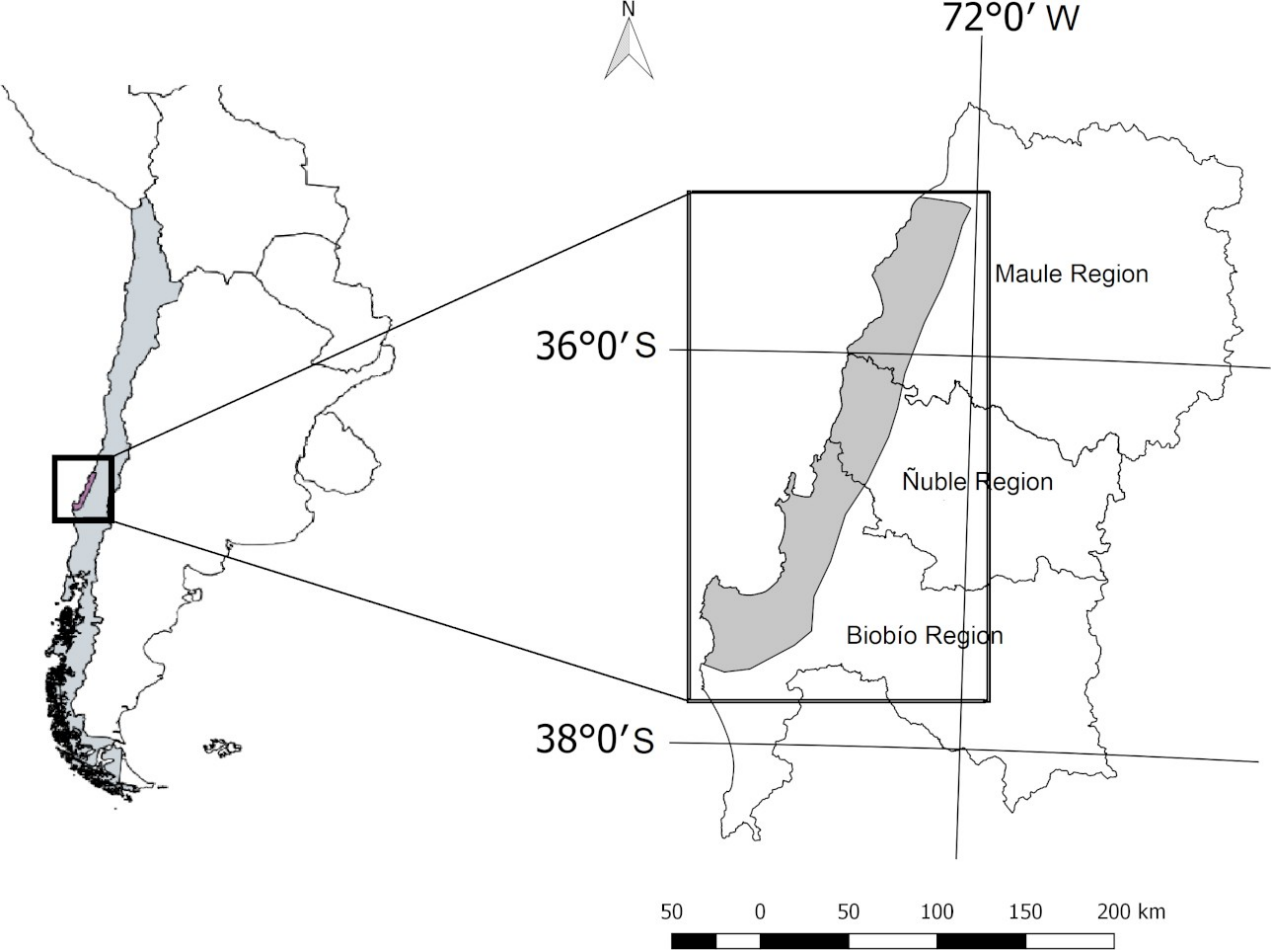
Study region. Coastal Range, Maule, Ñuble and Biobío regions.

Our study region covers approximately 950,000 ha, and is located in Coastal Range of Maule, Ñuble (recently declared as a new administrative region) and Biobio regions, between Putú and Coipué 35.2° S, 72.2° W in the north and, Lebu and Los Aromos 37.6°S, 73.2° W in the south (Fig 1). This region contains the oldest industrial forest plantations of Chile, which were established after the enactment of one of the first laws focused on forest conservation in 1931, and it is located in one of the world’s hotspots of biodiversity [39].

Before Spanish colonization, this region was inhabited by about one million people, who grew potatoes, beans, corn, among others, mainly in the valleys [40,41]. After Spanish colonization, the northern part of Biobío region was rapidly changed due to the introduction of livestock production [42]. After Chile’s independence in 1818, wheat production became strategically important, especially around 1850, when the region exported cereal crops to California and Australia during the gold rush [34,43]. This agricultural expansion caused a severe deterioration of native vegetation and soils, which is why vast areas of Maule, Ñuble and Biobío regions had moderate to high levels of erosion by the mid-20th century. Indeed, some provinces, such as Maule and Concepción, had more than 68% of their area eroded, especially in the Coastal Range [38]. To face this situation, the government decided to promote afforestation [44,45]. Although government subsidies were not restricted to any particular species, Monterey pine (*Pinus radiata*) ended up being the preferred species because of a combination of a fast growth rate and high-quality wood [34].

To quantify the past land uses we compiled a series of panchromatic aerial photographs available from the Chilean Military and Geographic Institute (IGM). The earliest photographs that we analyzed were from the Hycon flight taken in 1955 (1:70,000) over the northern part of the study region down to the Biobio river. From the Biobio river south, we analyzed photographs of the Organization of American States (OAS) project, of 1961 (1:50,000). We considered these two complementary sets of aerial photographs (1955–1961) as representing one moment, hereafter referred to as “1960”. We also used, as a support, maps that represented the distribution of forest plantations in the study region between 1945–1955 [37,38].

We georeferenced a total of 61 (1955) and 36 (1961) photographs using five Sentinel-2 satellite images from 2017 and a GIS vector dataset of road infrastructure obtained from Geospatial Infrastructure Data (IDE Chile) to identify ground control points. All data geoprocessing was carried out using QGIS 2.14 Essen [46], and all images were projected into WGS 84 18S UTM.

We also analyzed three Landsat MSS images (60 m resolution) for 1975 and three Landsat TM images (30 m resolution) for 1998 that covered the entire study region, and which we downloaded from Earth Explorer servers of the USGS (United States Geological Survey). Landsat images of 1985 were used to improve the level of certainty in the classification of points that were more difficult assign to a specific class, as an interdependent interpretation procedure [47]. Finally, we analyzed high-resolution satellite imagery available in Google Earth images, most of which were recorded in 2013–2014.

To assess land use change, we constructed a vector grid with 3-km resolution to obtain a point systematic sample of the study region, and we visually assessed land cover for each point and for each time (e.g. [48]). This grid was exported as kmz-file for point classification in Google Earth pro. Photo-interpretation was carried out using techniques that consider texture, tonalities, grey shades, and grain [49,50]. We used the same approach for the satellite images, but also the infrared band for MSS images, and infrared and Natural Color for TM images. In order to better interpret the MSS images, and to obtain an accuracy assessment for the MSS image interpretation, we also used 35 aerial photographs from 1978 and 1979, which stemmed from the CH 30 flight (1:30,000), and which we geometrically corrected and georeferenced using Sentinel images. Furthermore, we used historical information from reports (e.g. [37,38,51]) to help us understand the potential mechanisms behind land use changes. In addition, we conducted non-structured interviews with 22 local residents, most of them older than 70 years, to help us understand past land use trends, and to complement our literature review.

Although visual interpretation of images can be subjective and time consuming, we decided to use this technique because automatic classification has limitations when different types of images are analyzed (in this case, photographs and satellite images, simultaneously) [52,53]. Also, for each grid point, we recorded the land use cover information and a qualitative index of classification certainty (1: low, 2: medium, 3: high) (see S1 Table). In total, we assessed 1071 points for each of the three studied years. For 1960, no photographs were available for 22 points, thus limiting our dataset to 1049 point for that date. Once we had evaluated the land use class of each point for each date, we created transition matrices for 1960 –‘75, ‘75 –‘98, and ‘98–2014.

**Fig 2:**
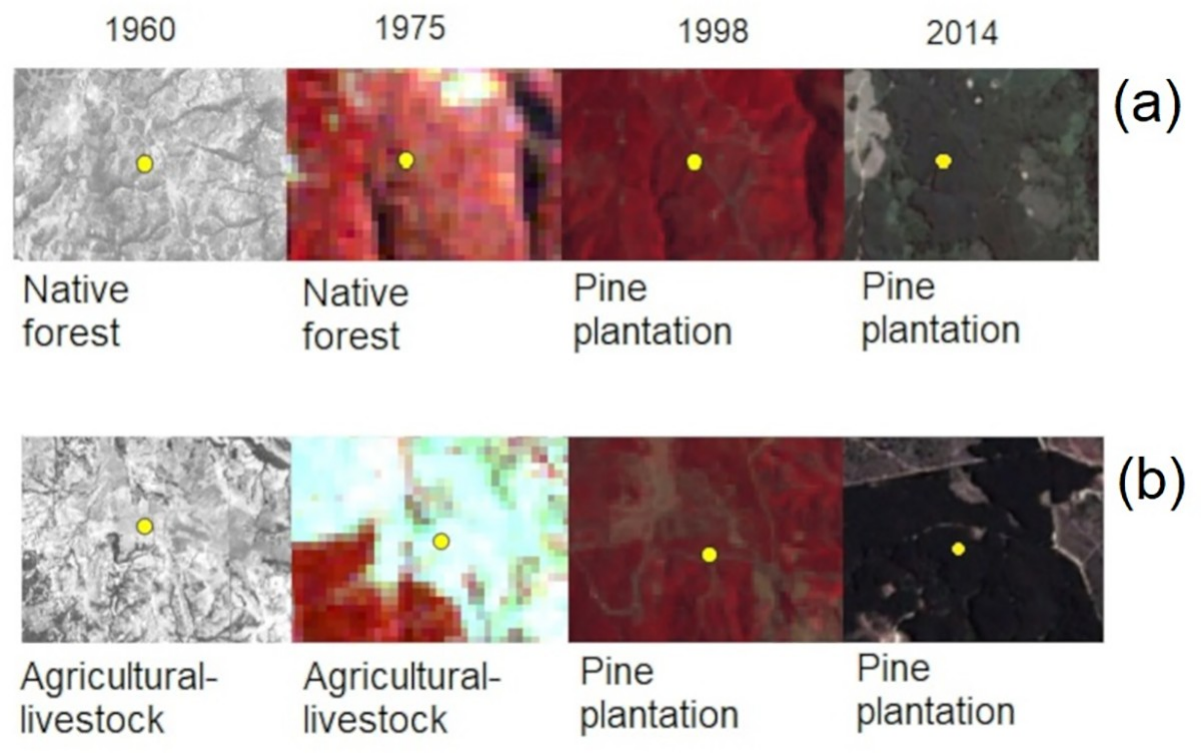
Example of point classification of land use in evaluated years. Changes from (a) native forest to pine plantation and (b) agricultural-livestock (including bare and fallow) lands to pine plantation.

**Fig 3:**
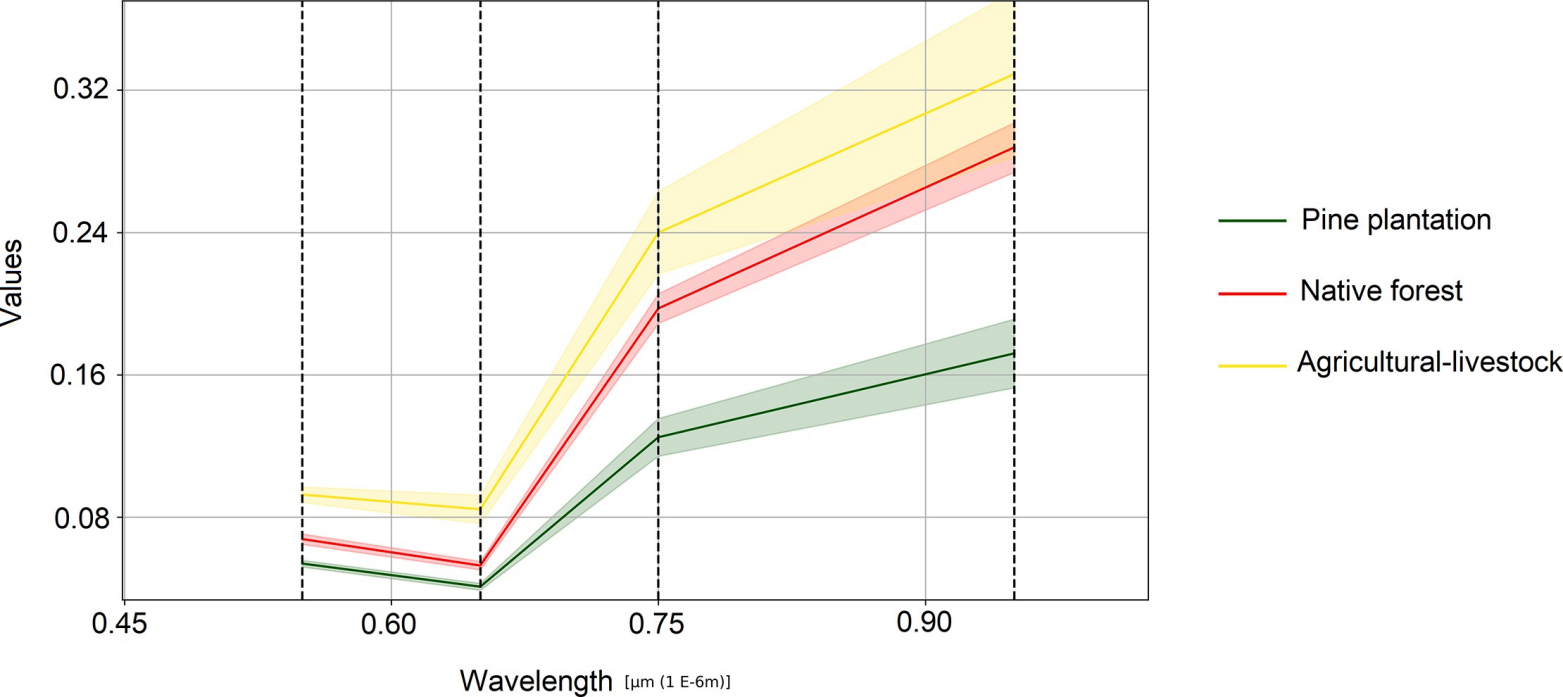
Reflectance curves (standard deviation in shaded areas) of the Landsat-2 MSS image.

We classified 10 different land use categories in our interpretation: agricultural-livestock lands, native forest, pine, degraded shrub, eucalypt, mixed uses, water bodies (rivers), unknown harvest (including those areas that were harvested at the time of the image obtaining, but we cannot ascertain its previous use), urban, other and no data points. However, in our analyses, we focused on four: native forest (including degraded forests), pine plantations, agricultural-livestock land (including fallow and bare land), and “others” (including all other classes) category. An example of point classification in different years and reflectance curves obtained from Landsat-2 MSS image for these categories are represented in Fig 2 and Fig 3, respectively.

In order to understand the role of different explanatory factors of the observed land use changes, we applied a logistic regression [54] to model the probability of change from a) native forest (0) to pine plantation (1), and b) agricultural-livestock areas (0) to pine plantations (1), for each transition period. Our explanatory variables were latitude, longitude, slope, Euclidean distance from each point to the nearest road, Euclidean distance to the nearest pulp mill, and the relative proportion of different land uses surrounding each point. Correlation among explanatory variables was tested to discard collinearity before performing generalized linear models. All variables were standardized and the best model was obtained by a forward and backward stepwise using the Akaike Information Criterion (AIC, [55]). We applied a Hosmer-Lemeshow goodness-of-fit test to the obtained models [56]. For all statistical analyses we used RStudio Version 1.0.136.

**Fig 4:**
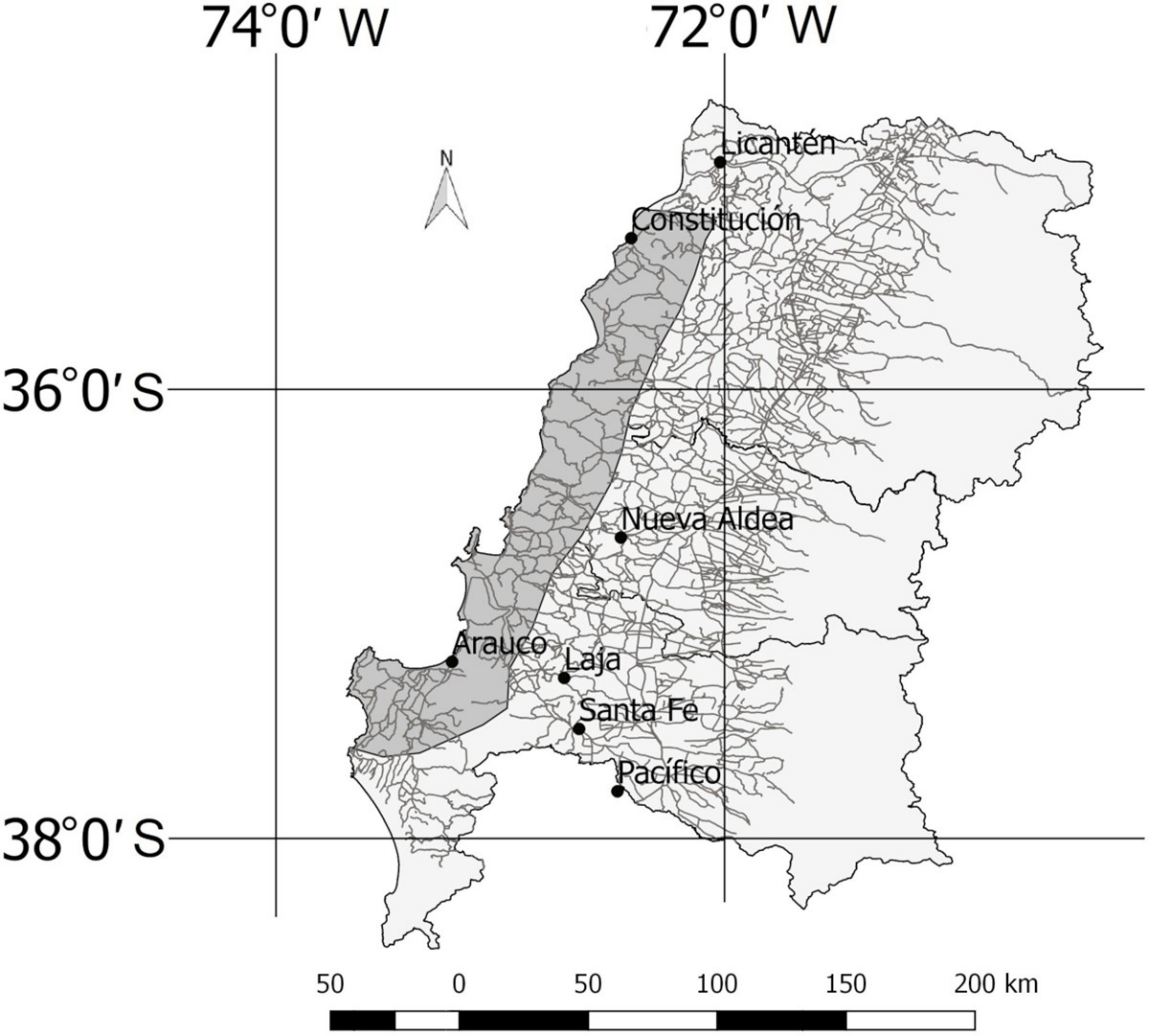
Pulp mills and road network location.

Latitude and longitude were calculated as UTM values for each point. To obtain slope values, we used the DEM generated by Aster imagery (product of METI and NASA), which we downloaded from USGS Earth Explorer server (https://earthexplorer.usgs.gov/). We calculated slopes values as percentages and the resulting raster layer was intersected with the shapefile layer of sample points to obtain their slope values. In order to obtain a road shapefile for each studied year, we modified the file for current one (Fig 4), and deleted the roads that did not appear in the respective photographs. The current shapefile did not include very small or temporary roads. Euclidean distance to roads was calculated using Grass GIS [57]. Distance to the nearest pulp mill was calculated for each period considering the facilities present at that time (Fig 4). Finally, for each point, we obtained a relative proportion of land use surrounding them by counting the number of points classified in each category among the 8 immediate neighboring points.

## Results

**Fig 5:**
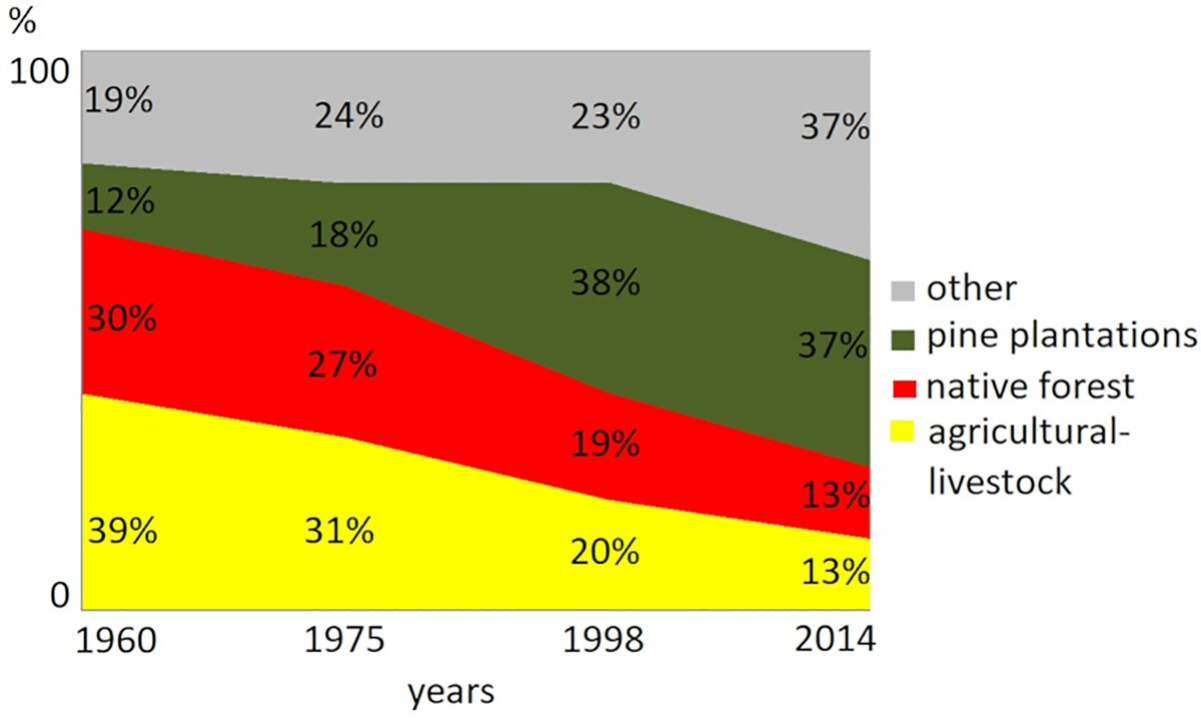
Percentage of points classified as pine plantations, native, agricultural-livestock and other land use each year.

**Fig 6:**
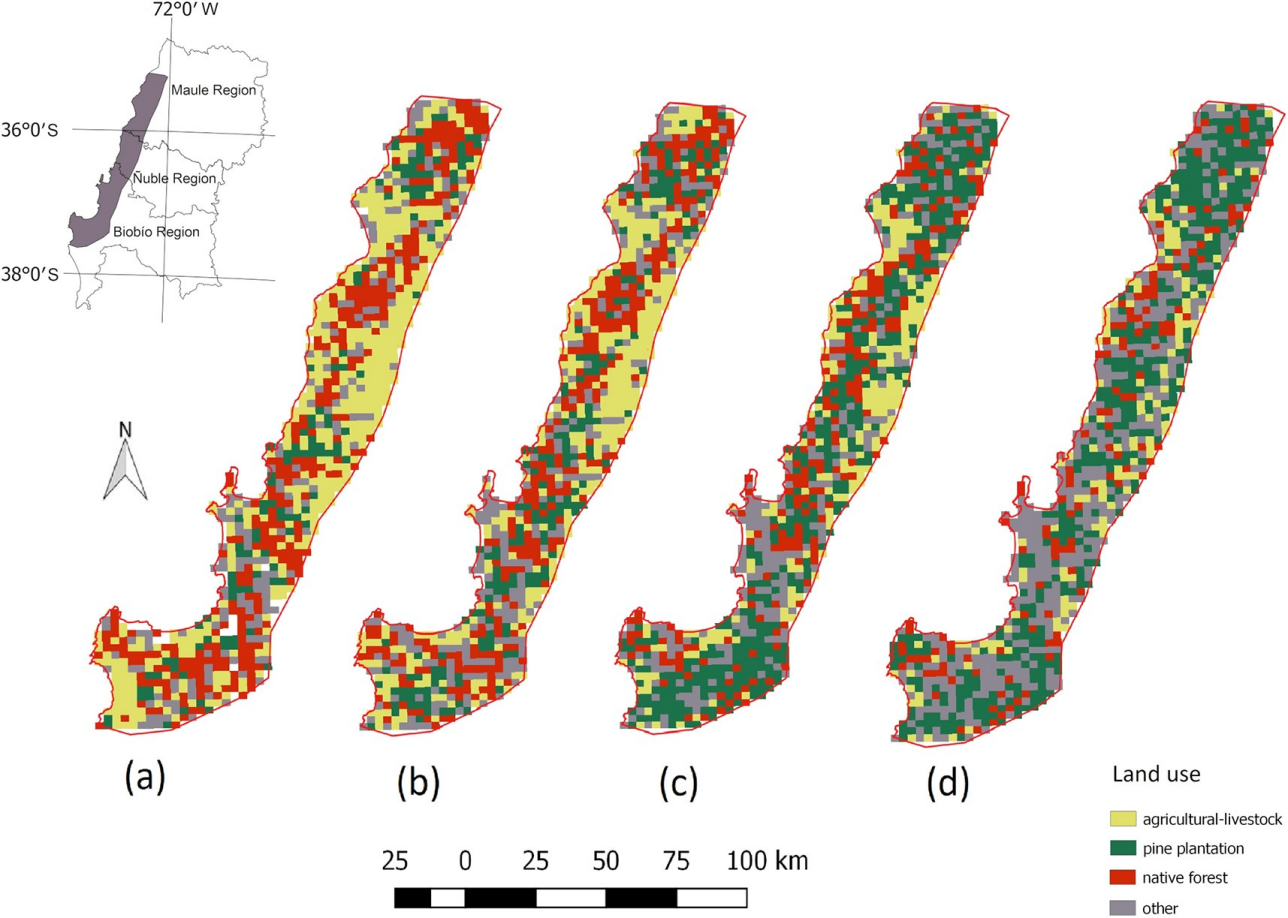
Representation of land use based on point classification. Land use for (a) 1960, (b) 1975, (c) 1998 and (d) 2014.

Image interpretation showed 39% of the study region was under agricultural-livestock use in 1960, while only 30% of the territory was covered with native forests, and 12% were already pine plantations (Fig 5). Pine plantations expanded rapidly from 1975 to 1998, increasing their cover from 18% of the sample points to 38%, accompanied by substantial reduction of native forest and agricultural-livestock lands, dropping from 27% and 31% to 19% and 20%, respectively. By the last studied year (2014), there was a small decrease in the cover of pine plantations (Fig 5), but native forest and agricultural-livestock cover were still declining, mainly in the central part of the study region (Fig 6). This reduction is due to the increase of eucalypt cover, because 18% of points that were pine plantations in 1998, were transformed into eucalypt in 2014 which is part of “other” category in Table 1. Accuracy of MSS images was around 60% (see S2 Table).

**Table 1:** Transition matrix for three periods.

**1960**	**1975**			
Land Use (%)	agricultural -livestock	native forest	pine plantations	other
agricultural-livestock	68 (3.78)	8 (0.44)	11 (0.62)	13 (0.72)
native forest	7 (0.40)	64 (3.53)	13 (0.72)	16 (0.90)
pine plantations	6 (0.31)	11 (0.62)	64 (3.57)	19 (1.06)
other	9 (0.49)	19 (1.09)	14 (0.75)	58 (3.22)
**1975**		**1998**		
Land Use (%)	agricultural -livestock	native forest	pine plantations	other
agricultural-livestock	57 (2.47)	7 (0.29)	21 (0.92)	15 (0.67)
native forest	2 (0.09)	47 (2.02)	40 (1.74)	11 (0.49)
pine plantations	1 (0.02)	4 (0.20)	78 (3.40)	17 (0.73)
other	6 (0.27)	16 (0.67)	25 (1.10)	53 (2.3)
**1998**		**2014**		
Land Use (%)	agricultural -livestock	native forest	pine plantations	other
agricultural-livestock	61 (3.79)	5 (0.30)	15 (0.95)	19 (1.21)
native forest	1 (0.06)	54 (3.38)	17 (1.07)	28 (1.74)
pine plantations	0 (0.03)	1 (0.06)	65 (4.05)	34 (2.10)
other	1 (1.10)	5 (0.30)	27 (1.69)	67 (4.16)

Values indicate the percentage of points of each category that changed from one land cover to another or remained unchanged between the specified years. In parentheses is the annual rate of change (%).

The largest change from native forests to pine plantations occurred from 1975 to 1998, with 40% (annual rate 1.74%) native forest being replaced by pine plantations (Table 1). Changes of agricultural-livestock lands to pine plantations occurred always at a lower rate than native forest with an annual change rate of 0.62%, 0.92% and 0.95% from 1960 to ‘75, ‘75 to ‘98, and ‘98 to 2014, respectively. An estimation of the area and their confidence intervals for each category in each evaluated year is in Table 2.

**Table 2:** Confidence intervals of the estimated areas for each category in each evaluated year with a critical value of 95%.

Estimated area (ha)	Years			
Category	1960	1975	1998	2014
Agriculture-livestock	372,553 ± 10,870	297,145 ± 8,224	189,419 ± 4,512	122,090 ± 2,435
Native Forest	283,679 ± 7,748	260,338 ± 6,928	184,032 ± 4,336	122,987 ± 2,460
Pine plantation	113,112 ± 2,183	176,850 ± 4,104	362,678 ± 10,528	356,394 ± 10,309
Other	192,112 ± 4,604	227,123 ± 5,778	225,327 ± 5,716	359,985 ± 10,434

Among all the changes that occurred from 1960 to 1975, 4.3% and 4.4% of all changes were from agricultural-livestock lands to pine plantations and degraded shrub, respectively (S3 Table). From 1974 to 1998, 10.8% of changes corresponded to the transformation from native forest to pine plantations. However, 6.5% of all changes also corresponded to the conversion of agricultural-livestock lands to pine plantations. Finally, the most important change from 1998 to 2014, representing the 6.8% of all changes, was the transformation of pine plantations to eucalypt plantations (S3 Table).

The probability of change from native forest to pine plantations from 1960 to 1975 showed a clear “contagion” pattern, in that this probability was significantly higher when the site had a higher proportion of pine plantations in the neighborhood (p<0.001) (Table 3). In the cases where two models had similar explanatory values (Δ AICc<2), both are presented in the table. Native forest surrounding native forest points also influenced the probability of change to pine plantations positively (p<0.05). From 1960 to 1975, the probability of native forest changing to pine plantations was higher in areas closer to pulp mills (p<0.05), and from 1998 to 2014, native forest surrounded by pine plantations were more likely to be converted into pine plantation (p<0.05).

**Table 3:** Effect of different variables on the probability of transformation of native forest and agricultural-livestock lands into pine plantations (logistic regression, binomial distribution).

				Surrounding cover					Hosmer and Lemeshow (GOF)	
Response variable	Slope (%)	Distance to pulp mill	Distance to roads	Agricultural–livestock	Native forest	Pine plantations	Δ AICc	AIC	X-square	p-value
NF 1960–1975		-0.42 (*)			0.41 (*)	0.65 (***)	0	210.58	2.56	0.96
NF 1975–1998							-	-	-	-
NF 1998–2014		.				1.14 (*)	0	64.66	6.66	0.57
AL 1960–1975	0.53 (***)				0.37 (*)	0.31 (*)	0	249.23	7.52	0.48
AL1975-1998							-	-	-	-
AL 1998–2014	0.87 (**)		.	.			0	113.03	11.62	0.17
AL 1998–2014	0.91 (**)		.	0.62 (*)		.	0.39	113.23	8.10	0.42

NF: Native forest to pine plantations, AL: Agricultural-livestock lands to pine plantations

GOF: Goodness of fit

p-value significance

(***) p<0.001

(**) p<0.01

(*) p<0.05

‘.’: Present in the model but without significance

**Fig 7:**
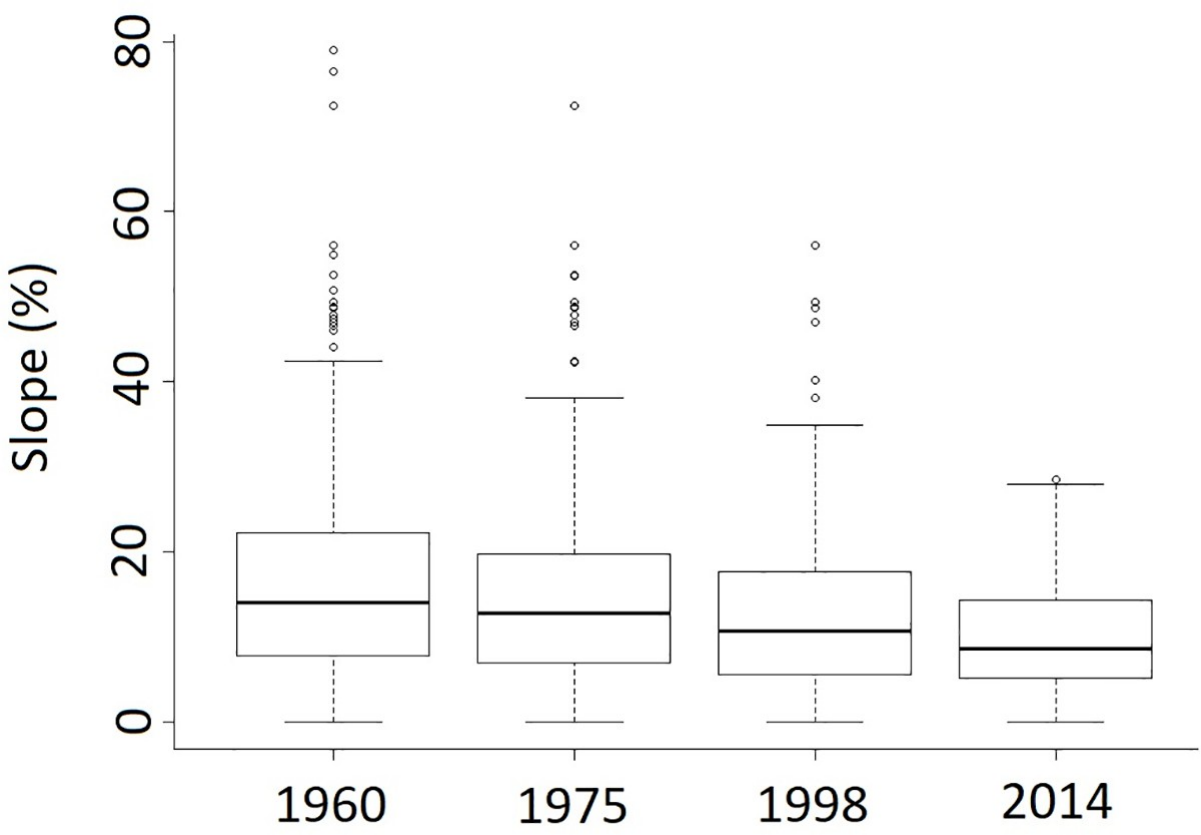
Slope of agricultural-livestock lands in different years in the Coastal Range of central Chile.

The transition from agricultural-livestock lands to pine plantations from 1960–1975 was more likely on steeper slopes (p<0.001), and where there were more pine plantations (p<0.05) and native forest (p<0.05) in the surrounding (Table 3). From 1998–2014, agricultural-livestock lands on steeper slopes were again significantly more likely to change to pine plantations (p<0.01). As a result, over time the average slope of agricultural-livestock lands decreased significantly (p<0.001, Kruskal-Wallis, Fig 7).

## Discussion

Native forest in the study region has been subject to strong land use pressures since middle of 19^th^ century due to the expansion of wheat production, and subsequently the development of intensive forestry since the 1950s. Pine plantations expansion has been favored by environmental, but also by economic conditions, and replaced native forests and also agricultural and livestock lands. However, the main causes for this expansion depended on the period analyzed, and on the prior land use. Neighboring of pine plantations, slope and distance to pulp mill, where in general strong predictors though.

Our results aligned well with what other authors had found in prior studies. The high rate of transformation of native forests into pine plantations from 1975 to 1998 agrees with the results of Nahuelhual et al. [24] who observed, in a part of our study region, that the highest expansion of forest plantations occurred from 1975 to 1990. Also, in that same study area, [23] found that a 53% of native forest present in 1975 was converted to forest plantations in 2000. Our results show that this rate was higher in degraded forests (Table 4), which suggests that native forest degradation can eventually lead to a transformation into forest plantation [24]. This high level of native vegetation loss occurred not only in our study area, but also in other parts of the Andean Range. More recently, southern regions have been subject to high pressure to convert native forest and agricultural lands to forest plantations [58].

**Table 4:** Percentage of total points with native forest or degraded forests at time i transformed in pine plantation at time j.

Period (i-j)	Native forest (%)	Degraded forest (%)
1960–1975	11	14
1975–1998	32	44
1998–2017	5	22

We found a native forest loss rate that was close to 2% per year due to the transformation into pine plantations between 1975 and 1990, which is similar to rates in countries with high deforestation such as Paraguay and Zimbabwe that had an average annual forest loss rate of near a 2% between 2010–2015 [3]. Furthermore, it has been found, in a portion of our study region, that deforestation rate reached a 4.5% per year from 1975–2000 [23], which is almost as high as the forest loss (5% per year) of Nigeria in the period 2010–2015 yr [3].

The progressive reduction of the agricultural-livestock lands (Fig 3), a great proportion of which were transformed into pine plantations, suggests that a part of the increase of these industrial forests followed the original “plan” of replacing degraded croplands. Although we cannot determine the level of degradation of open areas with the available information, the reduction in the average slope of open areas over time (Fig 5) suggests that plantations were first established in steep, marginal agricultural lands, as was observed by Nahuelhual et al. [24]. One of the objectives of Chile’s Forest Law of 1931 was to promote the establishment of forest plantations on eroded lands by means of tax exemptions and that could explain our results, but the current transformation could not necessarily be a replacement of marginal lands for agriculture as is proposed by the forest transition hypothesis [15], because good lands for agriculture were also under pressure for transformation into forest plantations [24].

The fact that from 1960–1975 the probability of native forests of being transformed into pine plantations was significantly higher when there were plantations in the surroundings suggests the existence of an imitation mechanism [59–61]. Thus, owners of forests close to pine plantations might have witnessed the economic advantages of growing pines, particularly in a time characterized by a strong incentive for the plantation industry, and little or no support for the management of native forests [34]. Also, replacement of native forests by crops was low during this period compared previous decades [45], most likely due to the decline of cereal prices [62].

From 1975–1998, the role of the studied factors on the transformation of native forests or agricultural-livestock lands into pine plantations was unclear. During this period, the highest rate of replacement of native forests into pine plantations occurred, likely promoted by subsidies established by the new Law-Decree 701 [23,63], similar to what occurred in Brazil, where deforestation was partly due to lower taxes and subsidies for agriculture or cattle production [64]. The lack of a clear spatial pattern of new plantation establishment might have been caused by economic incentives that were uniform across the study area and reduced the relative importance of location on the final decision to change the land use.

From 1998 to 2014, contagion appeared once again as a cause of the replacement of native forest into pine plantations. Currently, most owners in the region view native forests as an obstacle for economic development (Small owners, pers.comm.). Furthermore, their general experience is that neighbors that replaced their native forests with plantations are economically better off, although native forest replacement is illegal.

Legal efforts to protect forests and soils (Forest Decree Law 4,363 of 1931, Decree Law 701 of 1974 and Law 20,283 of 2008) have failed to save or enhance native forests in the studied region. During the five decades that we studied, almost 50% of the native vegetation present in 1960 was converted to pine plantations. The agrarian reform that took place between 1962 and 1973, and a military dictatorship between 1973 and 1990 were accompanied by economic changes that affected Chile’s native forests in a way similar to some Central-Eastern European countries before, during and after the Socialist regimes [65–67] where socio-economic and institutional changes affected ownership of lands.

Our results suggest that the expansion of forest plantations in Chile proceeded according to an agglomeration economy process. The legal support and promotion of planting trees for environmental improvements and the subsequent successful industry developed generated conditions for more investment in this sector, improving the existing roads and constructing new ones which increase profitability [68]. The contagion process observed is one of the traits of agglomeration economies [29] and if it is accompanied with a few environmental constraint and an open market including international demand, then competition and diversification of supply chain can cause even more other land uses to be transformed into plantations [31]. Clusters of economic activities have been seen in urban areas where there is a concentration of different production of goods and services that reduce cost, for example, in transportation, which allows industry to be more profitable and competitive attracting new investors [27].

Replacement of native forests by pine plantations started before the 1970s, which is the first sign that not only DL-701 favored its occurrence [69], but that there were also other variables strongly influencing this kind of transformation. During the 1950’s there were already approximately 180,000 ha of Monterey pine plantations in the Country, mostly of them in our study region and, to a lesser extent in the Central Valley of Biobío and Ñuble regions and the Malleco province of Araucanía region [37]. Although most of these plantations were established on degraded and abandoned lands that had previously been used for cereal production [34,43], the precise use that these lands were under at the time of afforestation is unknown to us. This uncertainty is reinforced by the fact that during our first study period native forests were already being replaced by pine plantations.

### Conservation implications

In the foreseeable future, a reduction in the replacement of native forest of the study region by industrial forest plantations can be expected. This is due to the decision of most large timber companies to formally protect the native forests left within their holdings, as part of the environmental certification processes in which they are all involved [70]. Besides, systems such as the Forest Stewardship Council certification require the restoration of forest areas equivalent or superior to those that were replaced before the certification system begun (1994, [71]). A drawback of this system is that it is only useful for big companies that export their products, but regulation of internal markets and also, small and medium size owner production, is not necessarily that efficient [70,72].

Although the transformation of agricultural-livestock lands into pine plantations can favor some forest wildlife when they are adult and well managed [73–75] there are attributes of native forests that are not present in forest plantations and therefore, native forest recover and habitat restoration in the study region is necessary to avoid future species loss.

Summer fires have increased since 2010 in magnitude [76], partly because a mega-drought has occurred in central Chile from 2005 to 2015 [77], both events probably related to climate change. The large fires that affected the region during the summer of 2017 [78] raised concern among residents about the potential role of forest plantations in spreading fire, due to their high water consumption and their spatial continuity. Besides, fires have further promoted the invasion by Monterey pines in areas of severely burnt native forests. If these events increase in frequency due to global warming [79,80], an additional pressure on native forests will come from invasion (and eventual replacement) by fire tolerant exotic species [81]. Because of the latter, calls have been made to reduce the cover of industrial forests.

Finally, total cover of pine plantations in the region has started to decline due to the expansion of *Eucalyptus globulus*. As shown by our data, in 2014, 18% of the area of pine plantations present in 1998 had been transformed into eucalypt stands. In Chile, the short-fiber paper and pulp industry has grown steadily due to the better quality of paper and shorter rotations of eucalypt plantations (less than seven years in some places) which makes them attractive compared to long fiber species [82]. This transformation is another challenge for future wildlife conservation mainly because there are uncertainties about the effect of eucalypt species plantations on wildlife in Chile.

## Supporting information

S1 Table Interpretation certainty level each year, considering percentage of points classified in the three arbitrary levels (low, medium, high). (PDF)

S2 Table Confusion matrix comparing interpretation of MSS image and aerial photograph based on 162 points. (PDF)

S3 Table Global transition matrix expressed as the percentage of all sample points that changed from one category to another, in the study region. (PDF)

S1 Data This file contains all information about sample point classifications that are the base for statistical analyses and creation of Figs 5–7. (XLSX)
