# Supplementary material for: Pine plantations and five decades of land use change in central Chile
Source: PLoS One. 2020 Mar 13;15(3):e0230193. doi: 10.1371/journal.pone.0230193 (PMC7069624; doi:10.1371/journal.pone.0230193)
Supplement: S1 Table — (PDF) [file pone.0230193.s001.pdf]

S1 Table. Interpretation certainty level each year, considering percentage of points classified in the three arbitrary levels (low, medium, high).

		Certainty level (%)		
Year	Land use	low	medium	High
1960	Agricultural-livestock	4.6	37.1	58.3
	Native forest	5.4	53.2	41.4
	Pine plantation	24.6	36.5	38.9
1975	Agricultural-livestock	34.7	50.8	14.5
	Native forest	40.0	51.1	8.9
	Pine plantation	26.4	35.5	38.1
1998	Agricultural-livestock	13.7	57.8	28.4
	Native forest	5.3	50.9	43.9
	Pine plantation	9.9	36.6	53.5
2014	Agricultural-livestock	0	5.1	94.9
	Native forest	0	1.9	98.1
	Pine plantation	0.3	8.1	91.7
