# Supplementary material for: Pine plantations and five decades of land use change in central Chile
Source: PLoS One. 2020 Mar 13;15(3):e0230193. doi: 10.1371/journal.pone.0230193 (PMC7069624; doi:10.1371/journal.pone.0230193)
Supplement: S2 Table — (PDF) [file pone.0230193.s002.pdf]

S2 Table. Confusion matrix comparing interpretation of MSS image and aerial photograph based on 162 points.

		MSS image 1975 yr				
**Photograph 1978 yr**	Category	Agriculture-livestock	Native forest	Other	Pine plantation	Total
	Agriculture-livestock	**26**	3	9	0	38
	Native forest	7	**41**	4	8	60
	Other	6	5	**8**	2	21
	Pine plantation	2	12	5	**24**	43
	Total	41	61	26	34	162

%					Average
omission	36.59	32.79	69.23	29.41	42.00
comission	31.58	31.67	61.90	44.19	42.33

Total accuracy	61,11
Total error	38,89
