# Supplementary material for: Pine plantations and five decades of land use change in central Chile
Source: PLoS One. 2020 Mar 13;15(3):e0230193. doi: 10.1371/journal.pone.0230193 (PMC7069624; doi:10.1371/journal.pone.0230193)
Supplement: S3 Table — (PDF) [file pone.0230193.s003.pdf]

S3 Table. Global transition matrix expressed as the percentage of all sample points that changed from one category to another, in the study region.

1960 (%)						1975 (%)						
Land-use	agr_liv	nat_for	pine	deg_shrub	euca	mixed	river	unk_harv	urban	other	ND	Total
agr_liv	26.3	3.1	4.3	4.4	0.2	0.0	0.0	0.1	0.3	0.1	0.0	38.7
nat_for	2.1	18.8	3.8	3.5	0.4	0.0	0.0	0.7	0.0	0.2	0.0	29.5
pine	0.7	1.3	7.6	0.7	0.2	0.0	0.0	1.3	0.1	0.0	0.0	11.8
deg_shrub	0.8	3.2	2.1	3.9	0.1	0.0	0.0	0.3	0.0	0.0	0.0	10.4
euca	0.0	0.0	0.2	0.0	0.2	0.0	0.0	0.0	0.1	0.0	0.0	0.5
mixed	0.0	0.0	0.0	0.0	0.0	0.1	0.0	0.0	0.0	0.0	0.0	0.1
river	0.0	0.0	0.0	0.0	0.0	0.0	2.1	0.0	0.0	0.0	0.0	2.1
unk_harv	0.0	0.0	0.0	0.0	0.0	0.0	0.0	0.0	0.0	0.0	0.0	0.0
urban	0.0	0.0	0.0	0.0	0.0	0.0	0.0	0.0	0.4	0.0	0.0	0.4
other	0.3	0.0	0.3	0.3	0.0	0.0	0.0	0.0	0.0	3.6	0.0	4.5
ND	0.7	0.7	0.2	0.1	0.0	0.0	0.0	0.2	0.0	0.2	0.0	2.1
Total	30.9	27.1	18.4	12.9	1.0	0.1	2.1	2.5	0.8	4.1	0.0	100.0

1975 (%)						1998 (%)						
Land-use	agr_liv	nat_for	pine	deg_shrub	euca	mixed	river	unk_harv	urban	other	ND	Total
agr_liv	17.6	2.1	6.5	2.4	0.6	0.1	0.0	1.0	0.5	0.2	0.0	30.9
nat_for	0.6	12.6	10.8	0.7	1.1	0.3	0.0	0.9	0.0	0.0	0.0	27.1
pine	0.1	0.8	14.4	0.3	0.7	0.2	0.0	1.8	0.1	0.1	0.0	18.4
deg_shrub	0.9	3.3	4.2	1.7	1.0	0.2	0.0	1.4	0.2	0.0	0.0	12.9
euca	0.0	0.1	0.4	0.0	0.4	0.0	0.0	0.2	0.0	0.0	0.0	1.0
mixed	0.0	0.0	0.0	0.0	0.0	0.1	0.0	0.0	0.0	0.0	0.0	0.1
river	0.0	0.0	0.0	0.1	0.0	0.0	2.1	0.0	0.0	0.0	0.0	2.1
unk_harv	0.3	0.2	1.3	0.0	0.1	0.0	0.0	0.7	0.0	0.0	0.0	2.5
urban	0.0	0.0	0.0	0.0	0.0	0.0	0.0	0.0	0.8	0.0	0.0	0.8
other	0.3	0.1	0.1	0.1	0.0	0.0	0.0	0.0	0.0	3.5	0.0	4.1
ND	0.0	0.0	0.0	0.0	0.0	0.0	0.0	0.0	0.0	0.0	0.0	0.0
Total	19.7	19.1	37.7	5.3	3.8	0.8	2.1	6.0	1.6	3.8	0.0	100.0

1998 (%)						2014 (%)						
Land-use	agr_liv	nat_for	pine	deg_shrub	euca	mixed	river	unk_harv	urban	other	ND	Total
agr_liv	12.0	0.9	3.0	1.0	1.9	0.3	0.0	0.3	0.3	0.1	0.0	19.7
nat_for	0.2	10.4	3.3	0.8	1.9	1.9	0.0	0.7	0.0	0.0	0.0	19.1
pine	0.2	0.4	24.5	0.6	6.8	1.2	0.0	4.0	0.0	0.1	0.0	37.7
deg_shrub	0.3	0.7	1.1	1.2	0.9	0.8	0.0	0.1	0.1	0.0	0.0	5.3
euca	0.0	0.1	0.7	0.0	2.4	0.2	0.0	0.4	0.0	0.0	0.0	3.8
mixed	0.0	0.1	0.1	0.0	0.0	0.6	0.0	0.1	0.0	0.0	0.0	0.8
river	0.0	0.0	0.0	0.0	0.0	0.0	2.1	0.0	0.0	0.0	0.0	2.1
unk_harv	0.1	0.1	4.4	0.1	0.8	0.0	0.0	0.3	0.0	0.2	0.0	6.0
urban	0.0	0.0	0.0	0.0	0.0	0.0	0.0	0.0	1.6	0.0	0.0	1.6
other	0.0	0.1	0.0	0.0	0.1	0.0	0.0	0.0	0.1	3.5	0.0	3.8
ND	0.0	0.0	0.0	0.0	0.0	0.0	0.0	0.0	0.0	0.0	0.0	0.0
Total	12.7	12.8	37.1	3.7	14.8	4.9	2.1	5.9	2.1	3.9	0.0	100.0

Classifications categories are: agricultural-livestock land (agr_liv), degraded shrub (deg_shrub), eucalypt plantations (euca), mixed uses (mixed) native forest (nat_for), no data (ND), other uses (other), pine plantations (pine), rivers, *unknown harvest (unk_harv) and urban.

* Unknown harvest classification corresponds to those harvested areas we cannot ascertain know if it is from previous agriculture or forest use.
